# Brain metastasis from an unknown primary, or primary brain tumour? A diagnostic dilemma

**DOI:** 10.3747/co.v16i1.308

**Published:** 2009-01

**Authors:** S. Campos, P. Davey, A. Hird, B. Pressnail, J. Bilbao, R.I. Aviv, S. Symons, F. Pirouzmand, E. Sinclair, S. Culleton, E. DeSa, P. Goh, E. Chow MBBS

**Affiliations:** * Rapid Response Radiotherapy Program, Department of Radiation Oncology, Odette Cancer Centre, Sunnybrook Health Sciences Centre, Toronto, ON; † Medical Oncology, Royal Victoria Hospital, Barrie, ON; ‡ Department of Laboratory Pathology, Sunnybrook Health Sciences Centre, Toronto, ON; § Department of Radiology, Sunnybrook Health Sciences Centre, Toronto, ON; || Department of Neurosurgery, Sunnybrook Health Sciences Centre, Toronto, ON

**Keywords:** Brain metastasis, glioblastoma multiforme, unknown primary, whole-brain radiation therapy, wbrt

## Abstract

Brain metastasis is increasingly common, affecting 20%–40% of cancer patients. After diagnosis, survival is usually limited to months in these patients. Treatment for brain metastasis includes whole-brain radiation therapy, surgical resection, or both. These treatments aim to slow progression of disease and to improve or maintain neurologic function and quality of life.

Although less common, primary brain tumours produce symptoms that are similar to those of brain metastasis. Glioblastoma, the most common malignant tumour of the brain, has a median survival of less than 12 months. Patients are often treated with surgical resection followed by radical radiation therapy and chemotherapy.

Here, we present 2 separate cases of lesions in the brain radiologically compatible with brain metastasis. In both cases, no primary cancer site had been established, and neurosurgical intervention was sought to obtain a pathologic diagnosis. Both cases were pathologically confirmed as glioblastoma. These cases demonstrate the importance of differentiation between brain metastases and primary brain tumours to ensure that the appropriate management strategy is implemented.

## 1. CASE PRESENTATION

### 1.1 Case 1

**FIGURE 1:**
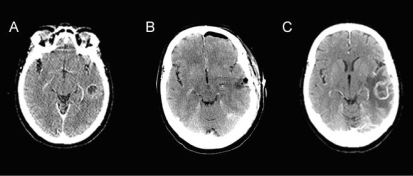
Post-contrast computed tomography (A) before resection demonstrates a part-solid, part-cystic, ring-enhancing lesion in the left temporal lobe; (B) immediately following resection demonstrates a resection cavity containing air and fluid without an enhancing abnormality (left frontal postsurgical pneumocephalus); (C) 2 months post-resection demonstrates expansion of ring enhancement within the left temporal lobe, associated with increased perilesion edema and mass effect.

A 63-year-old woman attended the emergency department with increased confusion and speech difficulty. She reported several episodes of aphasia occurring before this visit, with no history of headaches or seizures. Computed tomography (ct) imaging showed 2–3 lesions in the posterior temporal lobe, with accompanying edema [FIGURE 1(A)]. The multiplicity of lesions was reported to likely represent metastases.

**FIGURE 2:**
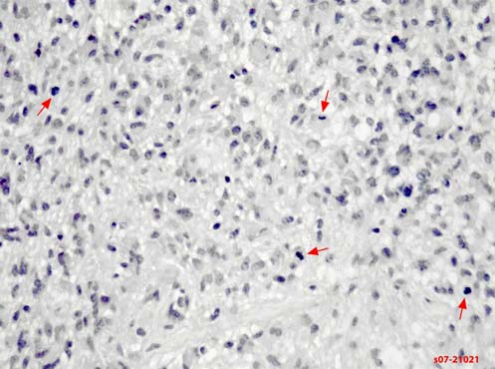
Pathology slide of glioblastoma multiforme (case 1). A section stained with hematoxylin and eosin shows frank atypia in this astrocytic tumour. Brisk mitotic activity is noted (arrows).

The patient was referred to a local medical oncologist, who began an extensive investigation for the primary cancer site. The patient was prescribed dexamethasone, which improved her symptoms significantly within 2 days. The patient was then referred for whole-brain radiation therapy (wbrt), pending primary cancer diagnosis. Investigation by the medical oncologist failed to reveal a primary cancer site. The patient was referred to a neurosurgeon for tissue diagnosis to guide the future use of systemic therapy. She underwent a left temporal partial craniotomy for excision of the tumour. Biopsy of the lesion showed evidence of glioblastoma multiforme (FIGURE 2).

**FIGURE 3:**
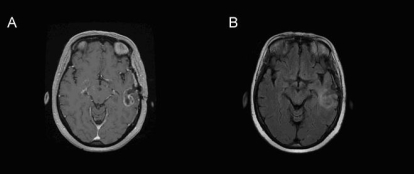
Volumetric (A) post-gadolinium T1 and (B) flair (fluid-attenuated inversion recovery) sequences 3 weeks post-resection, demonstrating a left temporal craniotomy, with a combination of underlying postsurgical changes and residual enhancing lesion surrounded by infiltrative edema.

Following the excisional biopsy, the patient began concurrent chemotherapy and radical radiation to the brain. Imaging by ct revealed evidence of residual tumour post-surgery [FIGURE 1(B)]. Radiotherapy was given postoperatively. A parallel opposed pair of beams was used to deliver 37.5 Gy in 15 fractions to a field encompassing the surgical bed, residual tumour, and edema with a 1-cm to 2-cm margin. A conformal boost based on CT and volumetric enhanced magnetic resonance imaging (mri) scan acquired during the third week of treatment [FIGURES 3(A, B)] followed. The boost volume was the enhancing volume as interpreted from the mri slices, without a margin. The 17.5-Gy boost was delivered in 7 fractions. The patient concurrently received temozolomide chemotherapy over 42 days, beginning on the first day of radiotherapy. She experienced some side effects from the chemotherapy, including pancytopenia and dysphagia secondary to mucositis and thrush.

About 2 months after her initial diagnosis, the patient presented again with aphasia and confusion. The ct conducted at this time showed progression of glioblastoma tumour in the left temporal lobe [FIGURE 1(C)]. The patient was referred to a palliative care physician in her community. Unfortunately, she passed away shortly thereafter: 3 months after initial presentation, and 2 months after tumour resection.

### 1.2 Case 2

**FIGURE 4:**
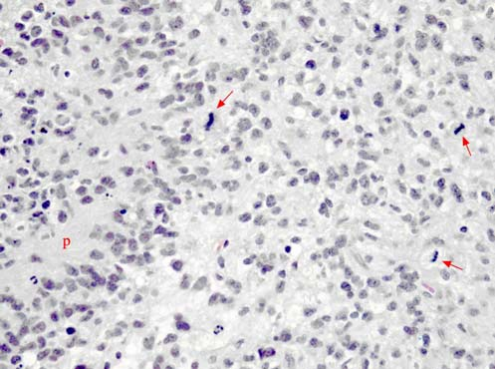
Pathology slide of glioblastoma multiforme (case 2). Some of the classic features of anaplastic astrocytoma are illustrated: nuclear pleomorphism, palisading necrosis (P) and brisk mitotic activity (arrows).

**FIGURE 5:**
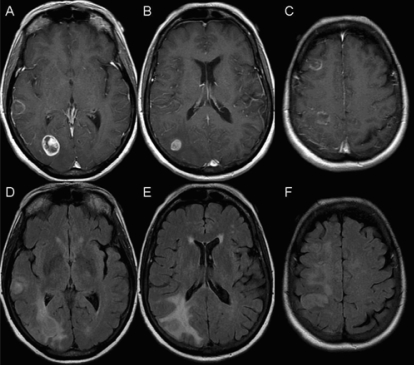
Magnetic resonance imaging slices showing glioblastoma multiforme (case 2). (A–C) Axial T1 post-gadolinium images and (D–F) corresponding axial flair (fluid-attenuated inversion recovery) slices at the same levels. Ring-enhancing masses can be seen in (A) the right temporal lobe, (A) right occipital lobe, (B) right inferior parietal lobe, (C) right frontal lobe, and (C) right superior parietal lobe. Under flair, hyperintensity can be seen around each lesion (D–F). Additional flair hyperintensity is seen intervening between the right frontal lobe and the right superior parietal lobe lesions (F).

A 62-year-old woman presented to the emergency department with severe right-sided headache and visual scotomata. Imaging by ct showed at least 3 ring-enhancing lesions in the right cerebral hemisphere of the brain, with associated edema. The multifocal nature of these lesions was more in keeping with metastasis than with primary malignancy. Because no evidence of a primary malignancy was found at any other site, the patient was referred to a neurosurgeon for biopsy of one of the more accessible tumours. The pathologic diagnosis proved to be glioblastoma multiforme (FIGURE 4). A mri investigation showed a total of 5 ring-enhancing lesions in the right hemisphere, with a mild amount of local mass effect and no midline shift [FIGURE 5(A–F)].

The patient underwent radiotherapy (50 Gy delivered using a parallel opposed pair of lateral brain fields) in 25 fractions over 5 weeks. Shortly after starting her course of radiotherapy, the patient was put on an adjuvant course of temozolomide that continued throughout her radiotherapy.

## 2. DISCUSSION

Glioblastoma and other gliomas are the most common type of primary brain tumour^1^, with an incidence of 5 in 100,000 in the U.S. population^2^. This frequency increases in older adults: the mean age of onset for glioblastoma is 53 years^3^. Patients with primary brain tumours commonly present with one or more symptoms that can include seizures (either partial or general in nature), increased intracranial pressure, or localized neurologic deficits such as weakness, motor problems, and aphasia^3^.

Although the predominant presenting symptom in brain metastasis is headache^4^, many patients have been reported to experience symptoms very similar to those seen with primary brain tumours. Specifically, estimates suggest that 50% of patients with brain metastasis have motor or language deficits^5^, and 10%–15% present with seizures^4^,^5^. Approximately 10% of patients with brain metastasis are asymptomatic, and the disease spread is identified through routine imaging^5^. Brain metastasis is evident in up to 40% of all cancer patients^6^–^10^, and overall, metastasis is more common than primary brain tumour, affecting 12 in 100,000 Americans^6^–^10^.

The preferred modality for detecting brain lesions, whether they originate from primary brain cancer such as glioblastoma or whether they are metastatic lesions originating from other primary sites, is mri with contrast administration^3^,^11^–^14^. Despite the accuracy of mri, ct imaging is often used when mri is either not available or not suitable for the individual case^7^,^14^,^15^. The argument has been made that the availability, efficiency, and cost-effectiveness of ct imaging make it the technique of choice in detecting brain lesions^16^. Pearl *et al.* suggested that non-contrast ct should be the initial investigation to detect any hemorrhage or calcification, followed by a double-contrast ct to identify lesions^16^. However, mri has the advantage of producing multi-planar images with higher contrast^15^. Images produced by T2 weighted mri are also more sensitive to smaller lesions^14^. Srikanth *et al.* reported a correlation between the histologic and morphologic features of tumours shown on both mri and ct imaging^15^.

Despite the advanced sensitivity of mri, 11% of patients with a brain lesion are given a false-positive diagnosis, based solely on mri, of either metastatic or primary cancer^17^,^18^. More recently, techniques such as perfusion mri have been used to differentiate primary gliomas and brain metastasis^19^. Often, diagnosis of brain lesions must be confirmed by pathology and histology examination, using excisional biopsy of the tumour^3^.

The appearance of lesions on mri or ct imaging will not always lead to a specific diagnosis. Even after examination of multiple images, diagnosis depends on many other factors. If the lesion is indicative of cancer, radiologists and oncologists must determine whether the lesion is a primary brain tumour, such as glioblastoma, or metastasis from a non-localized site elsewhere in the body. Approximately 80% of brain metastasis cases are diagnosed in patients who already have a known primary site of cancer^4^. This metachronous presentation makes the differential diagnosis less difficult. In cases of synchronous presentation, patients are diagnosed with a primary cancer around the same time that their brain metastasis is discovered. But before a primary site is found, it can be very unclear whether brain lesions are metastatic or not. Furthermore, for up to 15% of patients diagnosed with brain metastasis, the primary cancer site will remain unknown despite thorough investigation^5^. Positron-emission tomography imaging is a good tool for detecting an unknown primary when brain metastasis is suspected^20^.

Advances in radiologic imaging, including mri, have made the differential diagnosis between cancerous and non-cancerous lesions much easier. Before making a diagnosis of cancer, radiologists look for several features unique to lesions. However, the appearance of primary brain tumours and of brain metastases is often quite similar, making it difficult to distinguish between them, especially when considering only a single image. On mri slices, metastatic lesions often appear as small, well-defined, ring-enhancing lesions surrounded by edema^14^,^16^. They may also show central necrosis or hemorrhage, or both^16^. Gliomas are also found to have central necrosis surrounded by a ring of contrast enhancement; they also usually present with edema having mass effect^3^. A recent study comparing the appearance of brain lesions, including those caused by non-cancerous disease, found that 40% of ring-enhancing lesions on mri slices were caused by gliomas and that 30% were associated with brain metastasis^21^. Lesions caused by metastasis and gliomas were also found to have similar rates of hypo-intense borders on T2 weighted mri and to have similar rates of heterogeneous centres or central necrosis^21^.

New techniques in imaging are constantly emerging. One study showed that, as observed through perfusion mri, measurements of relative cerebral blood volume of the edema associated with metastasis were significantly lower than such measurements associated with glioma^19^.

Another factor that can significantly affect a differential diagnosis of brain lesions is the location and number of lesions. An estimated 20%–40% of cancer patients will develop multiple brain metastases^22^, as compared with the 30%–40% that will develop a single brain metastasis^23^. Solitary brain lesions have been found to be metastatic in only 15% of patients with an unknown primary site^24^. A recent study that compared brain lesion images found a significant difference between the prevalence of multiple lesions in brain metastasis (55%) and in glioma (23%)^20^. Furthermore, in glioma, non-localized multifocal lesions are rare; continuity between lesions occurs in all but 5% of cases^3^. Often, water diffusivity surrounding the tumour site is taken into account. Significantly different values for the apparent diffusion coefficient are reported in the region of flair (fluid-attenuated inversion recovery) hyperintensity surrounding ring-enhancing lesions caused by brain metastases and by gliomas^25^. For these reasons, multiple brain lesions are clearly much more suspicious for brain metastasis than are single lesions. However, to produce a diagnosis, care must be taken in the analysis of the radiologic evidence and of the symptoms. Ideally, pathology evidence should be obtained through biopsy for a confirmed diagnosis.

Symptoms and presentation of brain metastasis and glioblastoma can be quite similar, but patient prognosis can be quite different. The survival of patients with brain metastasis often depends on the primary site, but is usually about 12 weeks^6^. With wbrt, survival increases to 3–6 months in most patients^6^. Patients with completely untreated brain metastasis have a median survival of only 4 weeks^11^,^12^.

A recent clinical guideline outlined surgical resection followed by wbrt as the optimal treatment for brain metastasis^26^. Typically, surgical resection is considered only for patients with a single, surgically accessible lesion; with good performance status; and with a minimum of metastasis sites elsewhere^26^. Postoperatively, wbrt aims to reduce the incidence of tumour recurrence^26^. In patients with multiple metastases, wbrt is given, usually in doses of 30 Gy in 10 fractions or 20 Gy in 5 fractions^26^. Radiosurgical boosts may improve survival in patients with single lesions not eligible for surgery^26^. Chemotherapy remains an experimental treatment for brain metastasis, and supportive care is often a viable option. However, it is unclear which patients will benefit most from supportive care^26^.

Glioblastoma has one of the worst 5-year survival rates of all primary cancers^27^, with most studies reporting 4%–5% survival at 5 years^28^. One recent study found that the survival rates are actually overstated, with the true 5-year survival rate being closer to 2%^28^. The median survival for glioblastoma patients is usually less than 12 months^29^. Treatment can significantly improve survival, quality of life, and neurologic symptoms in these patients. Surgical resection of as much of the tumour as possible usually provides quick relief of symptoms and possibly a confirmed diagnosis, but the effect of surgical resection on survival is not confirmed^30^. Radiotherapy has been shown to significantly improve survival^31^ and is given in much higher doses than it is in brain metastasis patients. The maximum safe dose varies between 58–60 Gy in fractions of 1.2–2.0 Gy^3^. When this treatment is given instead of wbrt, it is usually concentrated at the site of the tumour^3^. Chemotherapy can be given instead of, or in combination with, radiotherapy. Alone, it has been reported to increase survival by about 6% and to provide a 15% reduction in overall risk of death^32^. Overall survival was improved in patients receiving radiotherapy with adjuvant chemotherapy as compared with patients receiving radiotherapy alone^33^.

In a recent phase iii clinical trial, the European Organization for the Research and Treatment of Cancer and the National Cancer Institute of Canada compared survival in glioblastoma patients treated with radiotherapy and concomitant and adjuvant temozolomide with survival in patients treated with radiotherapy alone. Results showed a significantly increased 2-year survival in the patients who received temozolomide (27%) as compared with the patients who received radiotherapy alone (10%)^34^. Temozolomide has proved to be a remarkably promising chemotherapeutic agent in glioblastoma, but the optimal dosing and route of administration of the drug have yet to be determined^34^.

## 3. CONCLUSIONS

Optimal treatment regimens in glioblastoma and in brain metastasis differ greatly. A good prognosis in both groups of patients depends on appropriate disease management. If the diagnosis is erroneous, neither group of patients will receive the care they need for improved survival and quality of life. Further, they may suffer unnecessary side effects induced by suboptimal treatment. Given the similar nature of both the symptoms and the presenting appearance of cancerous brain lesions, diagnoses are easily interchanged. It is therefore extremely important to exercise caution when diagnosing cancerous brain lesions. Many factors, including imaging, symptoms, number and nature of lesions, and patient history need to be taken into account. Pathologic evidence should be obtained whenever possible.
